# A new approach and first steps to strengthen trauma management and road safety in North Vietnam

**DOI:** 10.1186/1752-2897-2-10

**Published:** 2008-10-28

**Authors:** Uli Schmucker, Caspar Ottersbach, Matthias Frank, Luong Xuan Hien, Lajos Bogar, Axel Ekkernkamp, Dirk Stengel, Gerrit Matthes

**Affiliations:** 1 Ernst-Moritz-Arndt-University Greifswald, Department of Trauma and Orthopedic Surgery, Sauerbruchstrasse, 17575 Greifswald, Germany; 2 Unfallkrankenhaus Berlin, Department of Trauma and Orthopaedic Surgery, Warener Strasse 7, 12683 Berlin, Germany; 3 Thaibinh Medical University, 373 Ly Bon Street, Thaibinh City, Vietnam; 4 University Pecs, Department of Anaesthesiology and Critical Care, Ifjusag 13, 7624 Pecs, Hungary

## Abstract

**Background:**

In Vietnam, the number of road traffic accidents increased dramatically which is a major threat for the national health system. Reliable data on the magnitude of traffic accidents as well as the current management of victims is missing. Our multistep international cooperation project aims to (1) identify local needs and knowledge related to trauma management, to (2) assess basic behavioural patterns and attitudes of road users in order to (3) establish a school-based educational programme and trauma courses for doctors.

**Methods and results:**

As part of a European Union co-financed cooperation, two European and one Vietnamese university set up three action lines (Trauma and Emergency Courses, school-based education programs, public awareness campaigns). Specific contents of the activities were derived from a literature search, a questionnaire pilot-study and by panel consensus technique. After adjustment to local capabilities (equipment, infrastructure, etc.) these were implemented within a professional network of hospitals, schools, public and media institutions.

The literature research and questionnaire results from 1 000 young road users indicates that for pedestrian and two-wheelers accidents, low compliance with traffic regulations and high prevalence of risk-taking behaviour dominate Vietnam's road traffic environment. A school-based educational program (4 hrs/month) was set up using teachers who were trained on road safety issues. Also, major parts of the public awareness campaigns (i.e. broadcasts, media conferences) reflected these topics. From panel discussions and Delphi-technique, diagnosis and early treatment of severe head trauma and internal haemorrhage were identified as topics of highest interest for doctors therefore representing key topics of the Trauma and Emergency Courses.

**Conclusion:**

Knowledge on behaviour and attitudes of road users in Vietnam as well as on local infrastructure and effective networks is essential to establish sustainable and effective countermeasures. Our approach might serve as guideline for future small scale projects as it proved to be feasible, cost-effective but provided scientific base for immediate on spot activities.

## Background

The World Health Organization (WHO) reported 38 millions disability-adjusted-life-years lost (DALY) resulting from road traffic crashes (RTC) in 2002 [1]. Within this, 91,8% are allotted to low- and middle-income countries [1]. Amongst the 1.2 million RTC fatalities worldwide, approximately 25% were killed in South East Asia [1]. No other region worldwide has a similar incidence of RTC fatalities and registrations of new vehicles are expected to exponentially rise in near future.

In Vietnam, with increasing industrialization and urbanization, the number of registered motorcycles has increased from 1.8 million in 1992 to more than 11 million in 2003, representing approximately 95% of all registered vehicles [2]. The Ministry of Health published longitudinal data on motorcycle crashes between 1994 and 2002 showing an increase in the number of injured (from 14 174 in 1994 to 29 872 in 2002) and fatalities (from 4907 in 1994 to 12 956 in 2002) [3]. Nevertheless, the quality of national reporting systems in developing countries is often questionable as is the reliability of published data.

By common consent, prevention and improvement of medical care are well established, realizable and sustainable measures with lasting effects even in complex fields [4-9]. In this context Scott [10] emphasized "the need to identify the key causes of injury within the broad categories". Nevertheless, results and interpretation of findings from previous studies differ significantly with the specific country, region and setting being a probable cause. The statement of Sasser [11] that "each system must be defined by local needs and capacity and must be developed with due regard for local culture and health-care capacity" reflects this challenge that mandates in-depth and on-the-spot assessment involving local authorities.

With respect to trauma management, traffic and traffic crash reality on the scene, rescue logistics or medical teaching only few results from Vietnam with relevance for the current situation are available [5,7,12-16].

The goal of our project was (1) to capture local data on road users' attitudes and behaviour in a rural Vietnamese area and to (2) implement educational and public campaigns to improve trauma care and to increase awareness respectively. The study was performed in the City of Thaibinh (120 000 inhabitants), a city far from tourist tracks that is about to develop to a semi-urban regional centre. This paper focuses on our approach to assess local needs and derive measures for local application.

## Methods and results

### About the SAVE project

All research activities and prevention measures described in this paper were designed and realised within a project entitled SAVE. The SAVE-project was co-financed by the European Union's ASIA-Link fund. One German, Hungarian and Vietnamese university joined the consortium with 4–10 designated experts each. These were selected according to their specific expertise in the fields of traumatology, emergency medicine, traffic crash research, anaesthesiology and critical care, public health, epidemiology, psychology, health care and hospital management, administration, media and public relations. Within a 2-years period in 2005–2007 all activities were initiated and implemented under the supervision of the German team members. The following paragraphs describe our methods and how the results were transformed into specific measures.

### Enquiries and results

#### Mini-Delphi-technique

The Delphi technique is a systematic interactive forecasting method for obtaining forecasts from a panel of independent experts. The technique was adapted for use in face-to-face meetings and non-anonymous web-based conferences (mini-Delphi) [17]. Our mini-Delphi technique consisted of two rounds with web-based questionnaires and subsequent expert meetings (10 Vietnamese, 6 German, 4 Hungarian experts) which were held in the first 6 months of the project. The results in terms of items with highest relevance to the project are ranked in table 1.

**Table 1:** Results of mini-Delphi-process

Rank	Item
1	Crash vehicles: dominance of two-wheelers
2	Crash drivers: dominance of young males
3	Low compliance with traffic rules
4	Risk-taking driving behaviour accepted
5	Infrequent use of standardised algorithms

#### Questionnaire study

In order to generate a local database on road users' behaviour and attitudes an explorative questionnaire study was performed. A 23-item-questionnaire was distributed to and collected from n = 1000 students of the technical and teachers college of Thaibinh City. Within the questionnaire, 8 questions were relevant for development of key contents for the project activities. N = 764 complete questionnaires were included in the statistical analysis. Key results are presented in table 2. We found a mean age of 22.1 ± 3.3 years (43.5% male). Use of vehicle is dominated by bicycle (63.2%) and motorcycles (40.3%). Only 1.8% drive a car. A mean of 61.9 ± 17.8 km/h was found to be "a high driving speed". 12.3% of participants answered that they regularly drive despite alcohol or drug impairment. 17.2% are using a mobile phone "always" or "sometimes" while driving a two-wheel vehicle. Amongst motorcyclists, 48.7% "always" and 47.7% "sometimes" wear a helmet. As for our goal to address participants in an adequate setting we asked for sources relevant to information about road safety. Here, television (73.3%) and school (46.9%) were significantly more frequent than friends (26.7%), newspapers (26.6%), radio (26.3%) and family (24.7%).

**Table 2:** Key results of questionnaire study

Item	Results
Vehicle use	bicycle 63.2% motorcycle 40.3%
Violation against traffic rules	drink and drive 12.3% use mobile telephone: always/sometimes 17.2%
Source of information on road safety issues	TV 73.7% school 46.9%
Helmet use	always 48.7% sometimes 47.7%
Perceived "high driving speed"	mean value 61.9 km/h SD 17.8

#### Local adjustments

German and Hungarian teams participated in several missions in the Thaibinh province and city which gave substantial insight into local hospitals, teaching facilities and road traffic reality. Institutional capacity and infrastructure of the local medical university (and teaching hospital) and related province hospitals were of highest significance as these were designated to carry out the educational activities. Furthermore, the culture of teaching/learning and knowledge of English as foreign language were evaluated amongst the numerous designated partners in the hospitals. Last not least, arrangements with public institutions (i.e. the Peoples Committee of Vietnam), media and other authorities were made to secure the essential local support. Post agreement, a distinct time frame was drafted.

### Measures implemented

#### Trauma and Emergency Course (TEC)

TECs were implemented to support a sustainable improvement of medical care in the acute, injured patient. Within the project 5 TECs were carried out with 18–20 physicians each. A TEC consisted of 4 days with teaching units of 4 × 90 minutes or 8 × 45 minutes respectively. Table 3 presents key subject matters as derived from the Delphi-method, literature search and questionnaire study. Participants were recruited from hospitals of the Thaibinh province and city, thus coming from primary, secondary and tertiary level hospitals.

**Table 3:** Key contents "Trauma and Emergency Course"

Day	Key topic	Course content
1	Epidemiology,	RTA: mechanism, injury pattern
	Traffic accident research	Research: methods, scores, scales
		Prevention: methods, measures
2	Emergency diagnostics and treatment	Diagnostics: X-ray, ultrasound
		ER-algorithms
		Damage-control-principles
3	Severe head trauma	Clinical diagnostics and control,
		CT-diagnostics
		Indication for surgical intervention
4	Practical skills	Clinical examination
		Application cervical spine collar
		Motorcycle helmet removal
		Fracture stabilisation, bleeding control

Each TEC was evaluated by anonymous questionnaires; however a presentation of the evaluation results is not the subject to this report. In general, participants were very satisfied with the teachers, the teaching methods and the subject matters. On the other hand side, early diagnosis and emergency treatment of severe head trauma were found to be underrepresented in the first two courses. Therefore, based on informed consent amongst the project experts, the time frame of the course was restructured. Now, severe head trauma is listed as separate key subject matter on day 3 (see table 3).

Teacher-centred-teaching and short cases were used on days 1–3. An interpreter was present in all TECs as only few TEC participants had substantial knowledge of English language. For the practical training (day 4) the class was divided in small working groups of 3–4 members each. The groups then passed all skill stations (see table 3, day 4) by rotation.

Besides the "regular" TEC participants, 8 Vietnamese senior employees from the departments of surgery, emergency medicine, public health and epidemiology joined the TECs. These are defined as "tutors". All tutors participated in at least 2 TECs fulltime. In addition all tutors attended numerous further education courses in the German and Hungarian cooperation institutions. Here, they participated in the regular daily work on the ward, in the operation theatre and in the emergency department. They were trained in hospital management, critical incident reporting systems, medical education and teaching methods, clinical and traffic accident research. Last not least we offered courses in infection control, network computing and technology. Within several weeks the participants gained advanced background information. This should enable the tutors to independently carry out further TECs in Vietnam. Today, TECs are carried out not only in the Medical University of Thaibinh but also in many Thaibinh province and district hospitals.

#### School-based Educational Program (SEP)

Many prevention campaigns in the field of road safety do specifically address pupils. The rather confidential and familiar atmosphere in teaching institutions is supposed to enhance attention and support direct perception of subject matters. This can significantly increase the efficiency of measures. First of all, pupils are future drivers, future parents and "opinion-makers" and subsequently representing an attractive target group.

The SEPs pursued two main action lines. First, all-day courses for school teachers were organized in the Medical University of Thaibinh and in several province schools. Instructors for these courses were recruited from the project's tutors group. Key contents are listed below:

• Safe behaviour in road traffic (i.e. on cross roads)

• Significance of wearing motorcycle helmets and seat belts

• Socioeconomic relevance of road safety

• Teaching methods and strategies

Within the projects term a total of 120 teachers from 12 schools attended the courses. All participating school committed themselves to hold monthly "prevention sessions" of 4 hours each. In the meantime, the SEP is completely carried out by the Vietnamese cooperation partners.

#### Public Awareness Campaign (PAC)

Undoubtly, sustainable implementation of prevention measures requires adjacent campaigns effective in PR terms. Such campaigns are useful tools to link information to communicators/multiplicators therefore promoting general and purposeful broadcasting of content. On the other hand side, PACs support participation of opinion-makers and decision-makers. The SAVE PACs addressed several target groups. Press conferences were held in Germany, Hungary and Vietnam. Top representatives of the SAVE-project joined government and European Union representatives, media and representatives from institutions related to road safety to inform about contents and progress of the prevention measures.

In addition, promotion material (T-shirts, baseball caps, exercise books, etc.) was distributed amongst SEP participants. The exercise books were printed with comic strips showing a famous Vietnamese superhero promoting safe behaviour in road traffic (i.e. wearing motorcycle helmet). Lastly, a website was published as online educational and information tool .

## Discussion

Within an international cooperation project different programs in the field of medical education were developed, initiated and established in North Vietnam.

In the absence of large scale study results related to road traffic crash reality in Vietnam, key subject matters and contents had to be identified in the first project phase. The mini-Delphi-method, including panel discussions, is a well established systematic forecasting method to develop criteria within a panel of experts [17].

In consideration of basic demographic indices of Asian societies the panel expected young males being at highest risk for road traffic accidents. The clear dominance of males is undoubted; however, the association of age with risk of collision or injury remains unclear probably resulting from different study sites, cultural background, and methodological approaches [4,8,12,16,18-21].

The dominance of two-wheelers was shown for a rural Vietnamese community (93% of crash vehicles [12]). This is consistent with findings from other developing countries in Asia [16,20-23]. The results from our panel discussions and own results from young college students are consistent with these findings in so far as only 1.8% own or drive a car regularly but 24.4% of motorcycle drivers already experienced a collision.

A previous review of the literature on road traffic injuries in urban South East Asia found the highest percentage of children and adolescents injured were pedestrians (30%) [18]. Although these figures may not be representative for the rather rural setting of the Thaibinh area, our panel of Vietnamese experts confirmed that the majority of severe injuries in minor aged patients results from vehicle-to-pedestrian collisions. Consequently, crossing streets safely was a major content in the SEP. In addition, severe head trauma which is well known as the most frequent life threatening injury in both pedestrian and two-wheelers collisions therefore became one of the main contents of the TEMs.

Everybody who has ever experienced road traffic in South East Asia could witness that overloading bicycles, running red lights, driving on the wrong side of the road are widely prevalent in Vietnam and other South East Asian countries. Seat belt and motorcycle helmet usage are common indicators for compliance of road users. Hauswald et al. in Malaysia [24], Conrad et al. in Indonesia [22], Dandona et al. in India [25] and Nakahara et al. in Thailand [19] presented alarming figures in terms of using seat belts (40%) and wearing a helmet (25.1–55%) respectively. A previous roadside survey amongst n = 16 560 motorcyclists in Vietnam revealed a helmet rate of only 29.9% [13]. Similar figures, all indicating risky driving behaviour, were demonstrated in previous studies. Here, 36.5% out of n = 9948 motorcyclists [22] and 36.3% out of n = 683 motorcycle crashed drivers [18] were found to be driving under the influence of alcohol. Our own results were less sensational, but we expect a relevant bias from social desirability. One must conclude from these figures that lack of compliance is a fundamental problem of road traffic systems in South East Asia.

One major result from the mini-Delphi-technique was the assumption of an underdeveloped trauma system infrastructure, not only in the rural area of the province but also in the local university hospital. This is in agreement with the authors' experiences in Vietnam and a number of older investigations [4-6,26].

A hospital-based study from India found that no formal pre-hospital care was offered in 85% of the trauma patients [27]. Investigations in Thai, Iranian and Malaysian hospitals revealed, that only 0.7–1.4%, 10.7% and 12.3% respectively were transferred to hospital by qualified personnel and in special ambulance cars respectively [4,6,28] while a structured occupational training for emergency services does not exist [4].

In 2002–2003, Son et al. evaluated 23 institutions ranging from rural clinics to smaller district hospitals to larger city hospitals in and around Hanoi, Vietnam [15]. They assessed performance of the institutions pre (year 2002) and post (year 2003) use of the WHO-IATSIC Guidelines for Essential Trauma Care. This included 1-week trauma courses that were developed similar in concept to ATLS^© ^and Basic Trauma Life Support. The authors reported that "at baseline both human and physical resources were fairly adequate at the specialist-staffed city hospitals; intermediate at district hospitals; and largely not adequate at rural clinics". Post intervention they assessed a general improvement in certain life-saving procedures, however, the evaluation was subjective and based on self-reports. Furthermore, this study illustrates not only poor skills but severe limited physical resources like oxygen, basic airway equipment, cervical collars or IV fluids. Only city hospitals could provide few resources that are adequate by Essential Trauma Care-criteria. In a recent study in two areas including health care facilities of all levels in Vietnam, Son et al. identified shortages of mainly basic low-cost items and measures [7]. They emphasized wide use of continuing education courses for trauma care and more attention to trauma related curriculum in schools of medicine and nursing as cost-effective options to strengthen institutional capacity. This is in agreement with other studies that could determine a general association between equipment and level of qualification of personnel with patients [5,6], a fact that certainly contributes to the 50% of trauma patients that were recorded dead on arrival in the hospital [6].

While the results from Son et al. study gave evidence of feasibility and effectiveness of multimodal programs to improve trauma management, it also demonstrates the need for ongoing local assessment and interpretation of results from a local background. Our project site – though a city hospital and university hospital not very far from the Hanoi area – turned out to be completely different, without regular EMS for trauma patients, no 24 h service, no cervical collars or other basic equipment, ATLS^© ^or similar algorithms are unknown, computed tomography is not available. Our TEMs therefore focussed on clinical diagnostics and standardised algorithms in order to enable ED staff for early decision-making and avoid time loss in the early clinical phase. Of course, rapid assessment cannot compensate surgical intervention in many patients but it helps to improve quality of care, e.g. by defining criteria that mandate transfer to hospitals with advanced capabilities.

Already in 1993, Ali et al. found an improvement in trauma outcome following the implementation of ATLS^© ^programs in a developing country (Trinidad and Tobago)[29]. Nevertheless, use of standardized algorithms is still lacking. Results from a hospital based study from India showed that only one in eighteen hospitals had their ED medical staff trained in ATLS^© ^although trauma was responsible for 70% of ED visits [27].

An autopsy study from India [26] revealed that head trauma in 60% and abdominal/thoracic bleeding in 25% of fatalities are the most frequent causes of death after trauma. It's remarkable that the authors classified 67% of all fatalities as "preventable" or "possibly preventable". These can be attributed to misdiagnosis of a treatable injury (58%), time delay during transport (16%) and significant failure in treatment (18%). The authors highlighted two procedures every physician involved should be able to apply:

• rapid assessment of abdominal bleeding

• rapid onset of (surgical or conservative) treatment in the head injured patient

Next to severe head trauma, abdominal bleeding is one of the most frequent life threatening injuries in road traffic crash victims. Our intense teaching of diagnosis (i.e. ultrasonography) and treatment (i.e. damage control surgery-principles) do reflect the significance of these typical injuries. To the authors' view, focussing on typical, leading injuries like head trauma and abdominal bleeding is an efficient measure which promise a significant decrease of preclinical and clinical mortality.

In our own study sample of young college students a "high driving speed" of 61.9 km/h was documented. A roadside side survey in India found only 7% of motorcyclists driving faster than 50 km/h [25]. Given the underdeveloped infrastructure of Thaibinh city and province with heavy traffic in the city centres and bad road conditions, driving faster is nearly impossible. In this typical Vietnamese setting with rather low driving speed and dominance of vulnerable road users, a comparatively high rate of injuries which are treatable and survivable must be expected. Consequently, measures to improve trauma management promise a high effectiveness and efficiency.

Schools were named as important source of information related to road traffic safety in the questionnaire study and also in literature [9]. In their cluster analysis, Wong et al. analyzed different road safety policy strategies. Accordingly the target group "pedestrians" is addressed efficiently by public awareness campaigns with participation of non-governmental organisations. Broadcasting of relevant information in media and by distribution of promotion material was also found to be highly efficient. On the other hand side "motorcyclists" can be targeted by legislative/restrictive measures [9]. While the SAVE project cannot provide such measures, Wong et al. underlined that distribution of promotion material is the most effective measure to increase public awareness [9].

In Vietnam, teacher centred teaching is by far the most common teaching method which also allows students to copy the subject matters from the blackboard. Our intention to introduce new methods (i.e. problem-based-learning cases) was seen sceptically by the Vietnamese experts. In addition parallel interpretation restricts free choice of teaching methods. Except for the practical training sessions (day 4) and some case-scenarios containing accident mechanism, physiologic parameters and radiological files we limited teaching methods to teaching centred presentations.

Our project, although not a large scale investigation and intervention, emphasized the importance of the local focus, the local human and institutional factor. It aimed and succeeded at building a local network, identifying key criteria and developing low-cost countermeasures tailored to the specific needs of our partners. It set a solid base that enables all partners to jointly extend the efforts in near future.

## Conclusion

Lack of original, local data in all aspects of road traffic and trauma management mandates on spot and in-depth analysis. Only then, valid contents for project activities can be developed. Although laborious and costly, we strongly recommend such comprehensive approaches for both large and small scale projects. Viewed in the short term an upgrading of the local infrastructure on the level of industrialised countries is not realistic. Nevertheless, significant improvements in trauma care can result from low-cost measures if targeted to the vulnerable fragments in the continuum of trauma care. Next steps might include hospital based-trauma registries, accident, mortality or morbidity reporting systems and an upgrading of prevention measures on regional or state level.

## Competing interests

The authors declare that they have no competing interests.

## Authors' contributions

US led interpretations of findings and writing, assisted by MF. DS and CO developed methods and performed literature search, data entry, and statistical analysis. All field activities were led by MF, XLH, LB, AE, and GM. AE and GM supervised and steered the project. All authors were involved in designing the project, interpreted findings and reviewed the final draft of the manuscript.
